# Using network science to examine audio-visual speech perception with a multi-layer graph

**DOI:** 10.1371/journal.pone.0300926

**Published:** 2024-03-29

**Authors:** Michael S. Vitevitch, Lorin Lachs

**Affiliations:** 1 University of Kansas, Lawrence, KS, United States of America; 2 California State University, Fresno, Fresno, CA, United States of America; Education University of Hong Kong, HONG KONG

## Abstract

To examine visual speech perception (i.e., lip-reading), we created a multi-layer network (the AV-net) that contained: (1) an auditory layer with nodes representing phonological word-forms and edges connecting words that were phonologically related, and (2) a visual layer with nodes representing the viseme representations of words and edges connecting viseme representations that differed by a single viseme (and additional edges to connect related nodes in the two layers). The results of several computer simulations (in which activation diffused across the network to simulate word identification) are reported and compared to the performance of human participants who identified the same words in a condition in which audio and visual information were both presented (Simulation 1), in an audio-only presentation condition (Simulation 2), and a visual-only presentation condition (Simulation 3). Another simulation (Simulation 4) examined the influence of phonological information on visual speech perception by comparing performance in the multi-layer AV-net to a single-layer network that contained only a visual layer with nodes representing the viseme representations of words and edges connecting viseme representations that differed by a single viseme. We also report the results of several analyses of the errors made by human participants in the visual-only presentation condition. The results of our analyses have implications for future research and training of lip-reading, and for the development of automatic lip-reading devices and software for individuals with certain developmental or acquired disorders or for listeners with normal hearing in noisy conditions.

## Introduction

The methods and theory of the interdisciplinary approach known as network science—which draws from mathematics (where it is referred to as *graph theory*), sociology, computer science, physics, and other fields—are being used increasingly in a variety of disciplines [1], including the linguistic and cognitive sciences [2]. In this approach, *nodes* represent individual entities, and *edges* connect nodes that are related in some way to form a web-like *network* (or *graph*). The networks that are formed create something akin to a representational map, and should not be confused with other uses of the term “network” in the linguistic and cognitive sciences, such as artificial neural networks, connectionist networks, or parallel distributed processing models, which have been used to model various cognitive processes [3–5].

**Fig 1:**
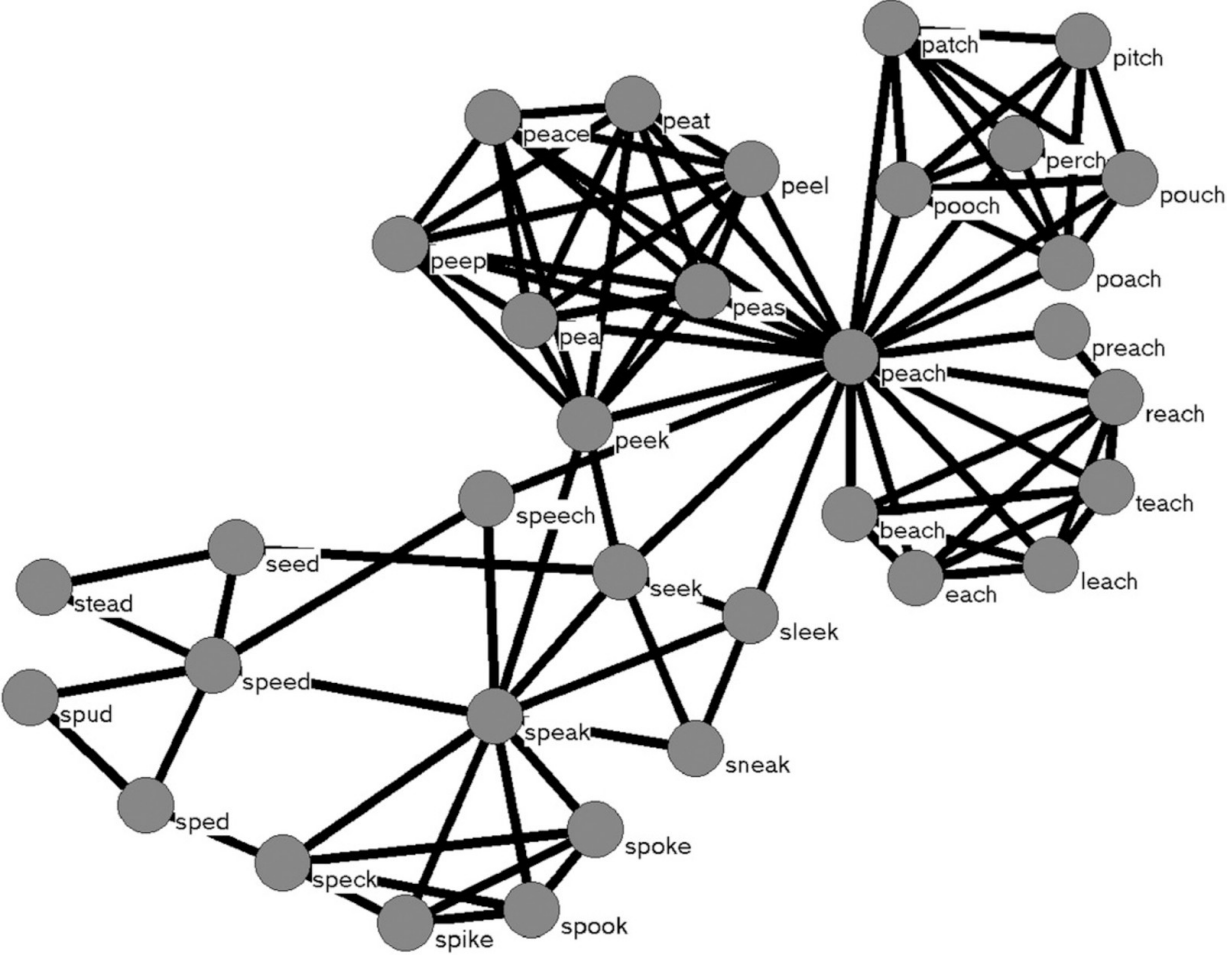
In this example, nodes represent (a subset of) words stored in the mental lexicon, and edges connect words that are phonologically similar to each other. Words are said to be phonologically similar if the addition, deletion, or substitution of a phoneme in a word forms the other word to which it is connected.

In the linguistic and cognitive sciences, the network science approach has been used to model, among other things, that part of memory known as the mental lexicon, which stores information about the words we know in a given language. In this application of network science, nodes represent the individual words one knows, and edges connect words that are semantically related [6], phonologically related [7], or orthographically related [8]. Fig 1 shows a subset of a network with words connected if they are phonologically related (as determined by the addition, deletion, or substitution of a single phoneme in one word to form another; for other ways of defining similarity between two word-forms see [9–11]).

The network science approach has significantly advanced our understanding of language processing in typical populations (for a review see [12]), as well as in children and adults with various types of developmental or acquired language disorders [13–17]. However, in the examples cited above, the networks contained a single layer of nodes to represent only one type of information (e.g., only phonological relationships among words).

More recent work in network science employs multi-layer graphs/networks to better understand how two or more different types of information might interact and affect processing. For example, a network with four layers was used to examine the explosive growth in the acquisition of words by young children [18]. In that network, nodes represented the same words in all four layers, but edges in different layers represented semantic free associations, synonyms, taxonomic relations, or phonological information. (Additional edges connected nodes in one layer to the same word/node in adjacent layers.) In another example, a multilayer network with semantic and phonological layers was used to better understand failures of word retrieval by people with aphasia [19].

In the present study we created a multi-layer network of word-forms that were phonologically similar to each other in one layer (as in Fig 1), and connected those words to another layer that represented how the words looked when spoken (i.e., during lip reading; [20]). That is, we transcribed the phonological representations of words like *pet*, *bell*, and *men* to a viseme representation of each word (all three words share the same visemes because they look alike when spoken; [21]) to examine how visual information about the lips and jaw might influence the perception of spoken words, and, conversely, how phonological information might influence visual perception of silently-spoken words.

The value of integrating visual information about the lips and jaws with the auditory information about the spoken word, especially under noisy conditions, was initially demonstrated in [22] where it was shown that word identification performance improved when listeners were able to see the lip and jaw movements of a speaker. More recent work has demonstrated that integrating visual and auditory information improves performance even when the words are clearly audible and intact [23]. Even deaf speech-readers who have minimal experience with spoken language show influences of lexical information (e.g., word frequency effects) when visually identifying spoken words [24].

Much research has demonstrated that the integration of auditory and visual information influences various aspects of spoken word recognition in a given language [25]. However, work by [26] demonstrated that the integration of auditory and visual information may also be involved in discriminating between two different languages. In the studies reported in [26] monolingual Spanish speakers and bilingual Spanish-Catalan speakers were asked to use only visual information about the speaker to determine whether Spanish or Catalan was being spoken. Both groups were able to discriminate whether Spanish or Catalan was being spoken (with monolingual Spanish speakers being less accurate than the bilingual Spanish-Catalan speakers). However, Italian and English speakers (who were unfamiliar with Spanish and Catalan) could not discriminate which language was being spoken from visual cues alone. In other words, the Italian and English speakers did not have access to—and therefore could not integrate—the phonological information associated with Spanish or Catalan to influence their decision about which language was being spoken using only visual information.

It is important for several reasons to increase our understanding of how visual and auditory information are integrated to influence word recognition. First, the World Health Organization projected that between 2015 and 2050, the proportion of the world’s population over 60 years of age will nearly double from 12% to 22% [27]. Common health conditions associated with aging include losses to hearing and to vision (e.g., cataracts), which could negatively impact word recognition. Given the expected increase in an aging population with conditions that may negatively affect word recognition, it is important to better understand how visual and auditory information are integrated during word recognition to develop new algorithms for hearing aids and other sensory prosthetics (e.g., cochlear implants) that can better compensate for the changes in visual and auditory perception that are associated with aging.

Second, the COVID-19 pandemic made the use of face masks much more common (in an attempt to slow the spread of the air-borne disease). Such face masks obscure both visual and auditory information associated with speech (e.g., [28]). Therefore, increasing our understanding of how visual and auditory information are integrated during word recognition could lead to the development of various communication strategies for speakers wearing a mask to compensate for the decrease of visual and auditory information associated with face mask use.

Finally, the COVID-19 pandemic contributed to increased use of audio-visual communication services (e.g., Microsoft Teams, FaceTime, Zoom). These software packages enable automatic real-time captioning to support individuals who are hard of hearing. Estimates of automatic transcription accuracy across various products ranges from 5 to 12 errors per 100 words [29]. Increasing our understanding of how visual and auditory information are integrated during word recognition could lead to improved accuracy in automatic transcription services by integrating the auditory signal of the speaker (as captured by the microphone found on many computer and mobile devices) with the visual information of the lips and jaw of the speaker (as captured by the camera found on many computer and mobile devices).

**Fig 2:**
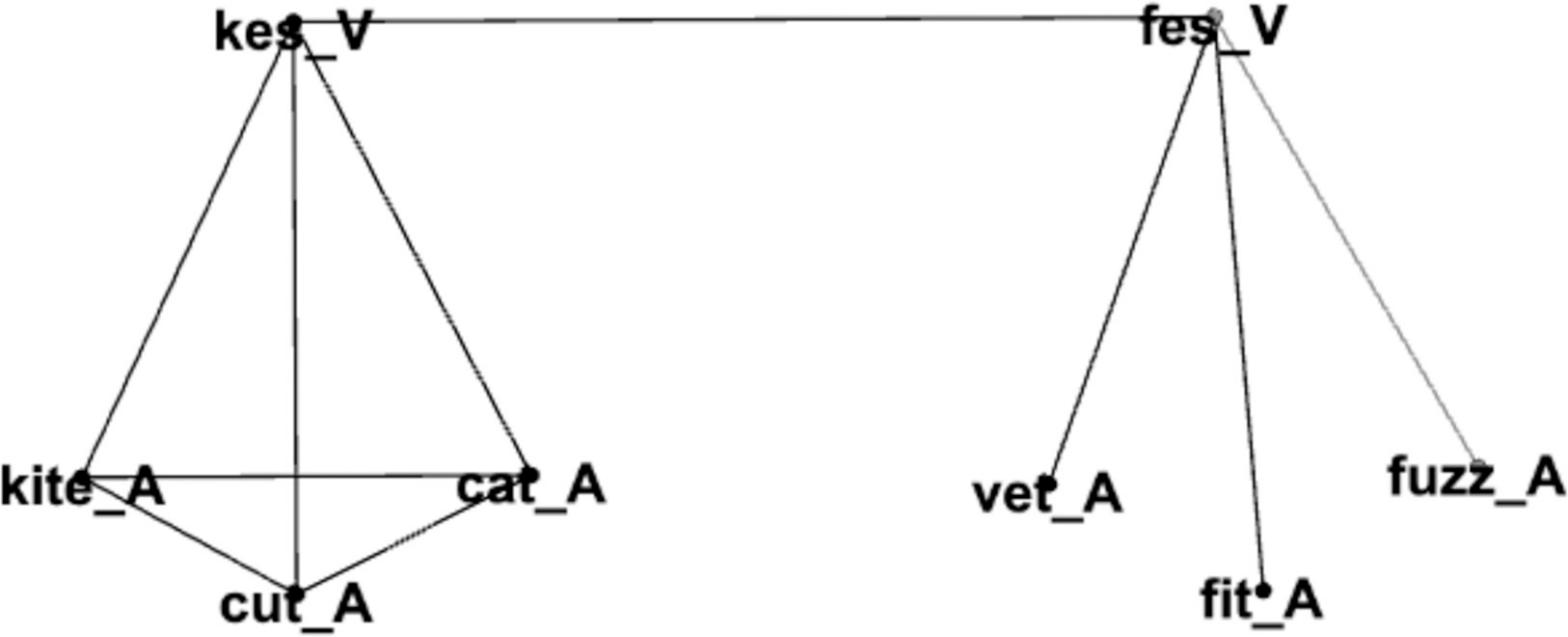
In this subset of the multilayer network, nodes in the bottom layer represent phonological word-forms (with “A” appended to the word), and edges in the bottom layer connect words that are phonologically similar to each other (based on the substitution of a phoneme in a word to form the other word to which it is connected). Nodes in the top layer represent viseme representations of words (i.e., how the word looks when lip-reading using a computer-readable transcription of viseme classes; with “V” append to those representations), and edges in the top layer connect representations that look similar to each other when spoken (based on the substitution of a viseme in a representation to form the other representation to which it is connected). Edges between the top and bottom layer connect phonological word-forms to their associated viseme representation. Different phonological word forms that map on to the same viseme representation are said to form a lexical equivalence class.

To help researchers in a variety of fields and disciplines examine audio-visual word recognition *in silico* we used the methods and techniques from network science to create a multilayer network containing two layers to represent auditory and visual information for a subset of common English words (see Fig 2). In the auditory layer of the network, nodes represented phonological word-forms, and edges connected words that were phonologically similar to each other (similar to the single-layer network in Fig 1). The number of edges incident to a node is referred to as *degree* (in the psycholinguistic literature the number of competitors is often referred to as *neighborhood density*; [11]) The visual layer contained nodes that represented how a word looks when spoken (i.e., as during lip-reading) by transcribing each word into its viseme representation. Edges in this layer connected viseme transcriptions that might be confused with each other (due to the substitution of a viseme). Another set of edges connected the phonological word-forms in the auditory layer to their associated viseme representations in the visual layer. Different phonological word-forms (like *pet*, *bell*, and *men*) that connect to the same viseme representation are said to form a *lexical equivalence class (LEC;* [21]). We refer to this multilayer network as the *AV-net* (AV stands for audio-visual).

We analyzed the network structure of the *AV-net* and conducted several computer simulations of a word identification task on the network. Although a central tenet of network science states that the structure of a network influences its function [30], we have relegated the analysis of the structure of the multi-layer network to the S1 File, so that we may instead focus on the performance of the network in the simulations and analyses summarized in Table 1 and described below.

**Table 1:** Summary of simulations and analyses.

	Network(s) used	Node(s) activated	Final activation obtained from	Effect being examined
**Simulation 1**	AV-net	Phono & Viz	Phono	Audio-visual condition in [31]
**Simulation 2**	AV-net	Phono	Phono	Audio-only condition in [31]
**Simulation 3**	AV-net	Viz	Phono	Visual-only condition in [31]
**Simulation 4**	AV-net	Viz	Viz	Inspired by effects in [26]
	V-net			
**Error analysis**	None (behavioral data)	N/A	N/A	Common error patterns

Note: AV-net = multilayer network with phonological and viseme layers; V-net = single layer network with only a viseme layer; Phono = phonological node; Viz = viseme node

Previously collected data from a word identification task asked different groups of human participants to identify a stimulus word under conditions of audio-only presentation, visual-only presentation, or with both audio-visual signals present [31]. In a word identification task participants are presented with a stimulus word and are asked to type on a computer keyboard the word that they perceived. Typically, the stimulus is only presented once, and participants are not under any time-pressure to respond. The typical dependent variable in a word identification task is percent word (or sometimes percent letters or phonemes) correct.

In the computer simulations in the present study the performance of the network was qualitatively compared to the behavioral data from the word identification task [31]. In Simulation 1 we modeled the condition in [31] where audio-visual signals were presented by activating the phonological node and the viseme node that corresponded to the stimulus word from [31]. After several time-steps elapsed (described in more detail in the Methods) we obtained the final activation value from the phonological node that corresponded to the stimulus word from [31].

In Simulation 2 we modeled the condition in [31] where only the audio signal was presented by activating the phonological node that corresponded to the stimulus word from [31]. After several time-steps elapsed we obtained the final activation value from the phonological node that corresponded to the stimulus word from [31].

In Simulation 3 we modeled the condition in [31] where only the visual signal was presented by activating the viseme node that corresponded to the stimulus word from [31]. After several time-steps elapsed we obtained the final activation value from the phonological node that corresponded to the stimulus word from [31]. In this simulation we again obtained the final activation value from the phonological node (instead of the viseme node) that corresponded to the stimulus word because in a word identification task participants are asked to respond with the word (not the jaw or lip gestures) that they perceived.

Simulation 4 was inspired by (but is not a direct replication of) the cross-linguistic results reported in [26]. Although numerous studies have demonstrated that the integration of auditory/phonological and visual/viseme information benefits word recognition within a given language [21–25], recall that in [26] monolingual Spanish speakers and bilingual Spanish-Catalan speakers, but not Italian and English speakers were able to use only visual information to determine whether Spanish or Catalan was being spoken. That is, monolingual Spanish speakers and bilingual Spanish-Catalan speakers had access to (at least some of the) phonological information for the Spanish or Catalan words that were being presented visually in the language identification task. However, Italian and English speakers did not have access to phonological information for either Spanish or Catalan to perform the language identification task.

In Simulation 4 we attempted to mimic having access to phonological information (as in the case of the monolingual Spanish speakers and bilingual Spanish-Catalan speakers) by using the AV-net used in Simulations 1–3. We attempted to mimic not having access to phonological information (as in the case of the Italian and English speakers) by removing the phonological nodes (and edges connecting the phonological nodes to the viseme nodes) from the AV-net to create a single-layer network that contained only viseme representations (which we refer to as the V-net). Rather than identify the language that was being spoken using only visual information as in [26], we attempted in Simulation 4 to examine how the presence/absence of phonological information might affect performance in a slightly different task—is the word lexically “easy” or “hard” to identify [11]—when only visual information is presented.

Finally, because much can be learned by examining the perceptual errors that people make [32], we analyzed the most frequent perceptual errors that human listeners reported in [31] during visual-only presentation. Note that the AV-net does not make “errors” in performance, making it impossible to directly compare simulated errors to actual errors made by human participants. Nevertheless, the context afforded by the AV-net may provide additional insight into how auditory and visual information are integrated during word recognition. Our materials are publicly available to enable researchers to explore new effects (as we do in Simulation 4) or to conduct preliminary tests *in silico*, before they invest time, money, and other resources into efforts related to the audio-visual integration of speech.

## Method

From the 19,340 English words used to form the phonological network in [7], we selected 1,070 monosyllabic words with a consonant-vowel-consonant (CVC) syllable structure. To ensure that most speakers of English would be familiar with the words, we selected items with a familiarity rating of 5 or greater on a seven-point scale, with 1 = *don’t know the word* to 7 = *know the word* [33]. We focused on a subset of words from the larger, previously used lexicon in part to keep manageable the computational demands (for network analysis and simulation) in the resulting multilayer network that we created. We focused specifically on CVC words in part because such words are commonly used in audiometric tests, such as the NU-6 (e.g., [34, 35]), and in tests of vowel perception where various vowels (V) are placed in the context of the consonants /h_d/ to form /hVd/ words like *heed*, *hid*, *hood*, and *head* [36].

The phonological transcriptions of these 1,070 words used the same computer-readable set of characters previously used in [37] to transcribe the full set of 19,340 words used in [7]. The phonological transcriptions of these 1,070 words became the nodes in the audio layer of the network with edges connecting words that differed by a single phoneme.

Table 2 shows the computer-readable set of characters used to transform the phonological representations in to the visemes that formed phoneme equivalence classes (adapted from Table 1 of [38]). Phonemes in the same phoneme equivalence class appear similar visually when they are spoken. The viseme transcriptions of the 1,070 words were used to create the visual layer of the network with 195 nodes (because many of the words appear similar visually when spoken this layer has fewer nodes than the audio layer) with edges connecting representations that differed by a single viseme. In addition, edges connected the phonological nodes in the audio layer to their associated viseme nodes in the visual layer.

**Table 2:** Phonemes and phoneme equivalence classes (i.e., visemes).

Phonemes (computer-readable)	Phoneme Equivalence Class
**Consonants**	
b, m, p	b
f, v	f
T, D	T
w	w
r	r
C, J, Z, S, d	C
t, s, z	s
k, g, h, G, y	k
n, l	n
**Vowels**	
I, I, e, Y, E, @, ^	e
R, o, O, U, u	o
a, c	a
W	W

Note. Phonemes in the same phoneme equivalence class appear similar visually when they are spoken.

We used the *R* package spreadR [39] to activate a selected set of words (described below) and assess how the diffusion of activation across the network affected simulated word identification. The following settings were used for the various parameters in each of the simulations. An initial *activation* value of 20 units was used for each stimulus word in the present simulations. Although *activation* = 100 units was used in the simulations reported in [40], this value is arbitrary. A smaller value was selected in the present simulations to reduce computational burden, thereby accelerating data collection.

*Decay* (*d*) refers to the proportion of activation lost at each time step. This parameter ranges from 0 to 1 and was set to 0 in the simulations reported here to be consistent with the parameter settings used previously (e.g., [41]).

*Retention* (*r*) refers to the proportion of activation that is retained in a given node after that node diffuses activation to the other nodes to which it is connected. This value ranges from 0 to 1 and was set to 0.5 in the simulations reported here. In [40] values ranged from 0.1 to 0.9 in increments of 0.1. Because the various retention values in [40] produced comparable results across retention values, we selected in the present simulations a single, mid-range value (0.5) for the retention parameter in order to reduce the computational burden, thereby accelerating data collection.

The *suppress* (*s*) parameter in *spreadr* will force nodes with activation values lower than the selected value to activation = 0. It was suggested that when this parameter is used a very small value (e.g., *s* < 0.001) should be used [39]. In the present simulations suppress = 0 to be consistent with the parameter settings used in [40].

*Time* (*t*) refers to the number of time steps that activation diffuses or spreads across the network. In [40] *t* = 10; however, in the present simulations *t* = 5. A smaller value was selected in the present case because as shown in Fig 3 of [39], activation values reach asymptote at approximately 5 timesteps, making additional timesteps uninformative. We selected in the present simulations a smaller value (*t* = 5) for the time parameter in order to reduce the computational burden, thereby accelerating data collection.

A typical “trial” in a simulation proceeds in the following way. At the initiation of the simulation (*t* = 0) the node (or nodes) corresponding to the stimulus (or stimuli), which we refer to as the *target node*, receives an initial activation of 20 units. At *t* = 1, the target node retains 50% of the initial activation (i.e., 10 units) and disperses the remaining amount (i.e., 10 units) equally to the nodes to which it is connected. For example, if the target node is connected to 2 nodes, each of those neighboring nodes would receive (10 ÷ 2 =) 5 units of activation from the target node. If the target node is instead connected to 5 nodes, each of those neighboring nodes would receive (10 ÷ 5 =) 2 units of activation from the target node.

At *t* = 2, the target node and the other nodes with non-zero activation (i.e., the neighbors of the target node) retain 50% of their initial activation. The remaining amount of activation in each node with non-zero activation is dispersed equally to the nodes to which they are connected. In the example of the target node having 2 neighbors, the target node would now have (5 + 2.5 + 2.5 =) 10 units of activation because the target node would retain 50% of the 10 units it previously had, but would also receive 50% of the activation from the 2 neighboring nodes. Note that if the neighboring nodes had additional neighbors that were not neighbors of the target node (referred to as 2-hop neighbors of the target node), then the amount of activation that the target node received from the 2 immediate neighbors would be less than 2.5 units because some activation would be dispersed to the 2-hop neighbors as well as to the target node.

This process of retaining some activation, dispersing the remaining activation to connected nodes, and adding any activation received from nodes to which one is connected repeats until *t* = 5. This results in activation being broadly dispersed through the network among neighbors, 2-hop neighbors, etc. The dispersion of activation in such networks is more similar to simple diffusion models in physics than to the more complicated spreading activation models often seen in the linguistic and cognitive sciences, which might include facilitatory as well as inhibitory connections, decay of activation (although see the *d* parameter above), unidirectional spreading of activation, various thresholds, nodes with different resting activation levels, etc. Despite the simplicity of the diffusion of activation over the type of network employed in the present study, quite complex behaviors have been computationally reproduced via simulations (e.g., [39–41]).

The stimulus words used to assess the performance of the network were selected from [31]. The database reported in [31] contained 300 *easy-hard* words [11], and the collection of the data contained in [31] was reviewed and approved by the institutional review board at Indiana University. Participants in [31] (all of whom were 18 years of age or older) provided informed written consent witnessed by a research assistant. None of the data reported in [31] contained information that could identify individual participants.

Lexically “easy” words occur frequently in the language and are phonologically similar to few other words, which makes them “easier” to identify than lexically “hard” words, which occur less often in the language and are phonologically similar to many other words [11]. From the set of 300 words in [31], we selected 76 words (38 easy words and 38 hard words) that had a CVC syllable structure. To control for possible differences in the phonemes/visemes that may be typically found in lexically easy words versus lexically hard words, the initial consonant found in the easy words was matched to the initial consonant found in the hard words.

At the end of 5 timesteps in the simulations we documented the activation level of each of the stimulus words. Higher levels of activation correspond to “good” performance and lower levels of activation correspond to “poor” performance in a psychological or linguistic task. For tasks where reaction time is the dependent measure higher levels of activation correspond to rapid reaction times, whereas lower levels of activation correspond to slow reaction times. For tasks (such as the word identification task used in the present case) where accuracy is the dependent measure higher levels of activation correspond to more accurate responses, whereas lower levels of activation correspond to less accurate responses. The interpretation of activation levels adopted in the present study is consistent with the interpretation of activation levels in other computational models of language processing [40, 42].

To simulate the word identification task in which audio-visual signals were both presented, the phonological word form corresponding to the easy-hard word received 20 units of activation, and the viseme representation corresponding to the easy-hard word received 20 units of activation (*Simulation 1*). After 5 time steps the final activation level was obtained from the phonological word form that corresponded to the easy-hard stimulus word to coincide with the word that human participants identified in the psycholinguistic task in [31].

To simulate the word identification task where only the audio signal was presented, only the phonological word form corresponding to the easy-hard word received 20 units of activation (*Simulation 2*). After 5 time steps the final activation level was obtained from the phonological word form that corresponded to the easy-hard stimulus word.

To simulate the word identification task where only the visual signal was presented, only the viseme word form corresponding to the easy-hard word received 20 units of activation (*Simulation 3*). After 5 time steps the final activation level was obtained from the phonological word form that corresponded to the easy-hard stimulus word.

In Simulation 4 we examined how access to phonological information might influence the visual recognition of silently spoken words by activating (with 20 units of activation) the viseme word form corresponding to the easy-hard word in the AV-net. To examine how not having access to phonological information might influence the visual recognition of silently spoken words we created a single-layer network that contained only viseme representations (the V-net). In the V-net, the viseme word form corresponding to the easy-hard word received 20 units of activation. After 5 time steps the final activation level was obtained from the viseme word form in the AV-net and in the V-net that corresponded to the easy-hard stimulus word.

To determine if statistically significant patterns in the errors produced during the visual recognition of spoken words could be observed (e.g., the initial phoneme tends to be misperceived more often than the final phoneme, etc.), we examined the most frequently reported incorrect response for each of the 76 easy-hard CVC words that were presented in [31]. Because the AV-net does not make “errors,” we were not able to directly compare performance in the network model to human performance in [31].

## Results

Analyses comparing the performance of the network to the performance of human participants in the word identification task (reported in [31]) on the 76 easy-hard words were performed with *JASP* (Version 0.16.3; [43]). Independent samples *t*-test were used to compare the activation values for the lexically easy and the lexically hard words in the AV-net when the final activation values were obtained after 5 time-steps. Because performance is expected to be “better” for lexically easy words compared to lexically hard words the *t*-tests are one-tailed unless specified otherwise.

In Simulation 1, where phonological and viseme nodes were both activated to simulate the word identification task in which audio-visual signals were both presented, the phonological nodes corresponding to easy words had higher activation values (i.e., were identified more accurately; *mean* = 1.49 units, *sd* = .23) than the phonological nodes corresponding to hard words (*mean* = 1.37 units, *sd* = .10). This difference was statistically significant (*t* (74) = 3.20, *p* < .01) and is qualitatively similar to the performance of human participants in the word identification task reported in [31].

In Simulation 2, where only phonological nodes were activated to simulate the word identification task in which only the audio signal was presented, the phonological nodes corresponding to easy words had higher activation values (i.e., were identified more accurately; *mean* = 1.24 units, *sd* = .14) than the phonological nodes corresponding to hard words (*mean* = 1.15 units, *sd* = .06). This difference was statistically significant (*t* (74) = 3.77, *p* < .001) and is qualitatively similar to the performance of human participants in the word identification task reported in [31].

To determine if the AV-net captured the “boost” in performance that the integration of visual and phonological information gives to word identification compared to relying on only phonological information (as demonstrated in [22, 23]) we compared the final activation values from Simulation 1 (where both phonological and viseme nodes were activated, but output was obtained from the phonological nodes) to the final activation values from Simulation 2 (where only phonological nodes were activated and output was obtained from the phonological nodes). A two-tailed paired-samples *t*-test (*t* (75) = 24.67, *p* < .001) shows that the integration of phonological and viseme information as obtained from the phonological nodes in Simulation 1 had higher activation values (i.e., were identified more accurately; *mean* = 1.43 units, *sd* = .19) than phonological-only information as obtained from the phonological nodes in Simulation 2 (*mean* = 1.20 units, *sd* = .12).

In Simulation 3, where only viseme nodes were activated to simulate the word identification task in which only the visual signal was presented, the phonological nodes corresponding to easy words had higher activation values (i.e., were identified more accurately; *mean* = 0.25 units, *sd* = .10) than the phonological nodes corresponding to hard words (*mean* = 0.21 units, *sd* = .06). This difference was statistically significant (*t* (74) = 1.93, *p* < .05) and is qualitatively similar to the performance of human participants in the word identification task reported in [31].

In Simulation 4 we examined how being “cut-off” from phonological information might affect processing (as in [26]). We constructed a one-layer network containing just the viseme nodes, with edges placed between viseme word-forms that differed by a single viseme (i.e., the V-net). The visemes that corresponded to each of the 76 easy-hard words were activated (with the other activation parameters remaining the same as those used in Simulations 1–3), and after 5 times-steps the final activation values were obtained from the viseme nodes. With no access to phonological information the viseme nodes corresponding to easy words in the V-net had comparable activation values (*mean* = 1.26 units, *sd* = .07) to the viseme nodes corresponding to hard words in the V-net (*mean* = 1.25 units, *sd* = .04). This difference was not statistically significant (*t* (74) = 1.35, *p* > .05).

In the “intact” AV-net we activated the visemes that corresponded to each of the 76 easy-hard words, and after 5 times-steps the final activation values were obtained from the viseme nodes. In this case, the viseme nodes corresponding to easy words had higher activation values (i.e., were identified more accurately; *mean* = 1.14 units, *sd* = .10) than the phonological nodes corresponding to hard words (*mean* = 1.07 units, *sd* = .05). This difference was statistically significant (*t* (74) = 3.52, *p* < .001).

Although the AV-net does not make “errors” in performance (making it impossible to compare simulated errors to actual errors made by human participants), there is still much to be gained by examining in various ways the perceptual errors that human listeners reported in [31] in the context of the AVnet [32]. Because performance was near ceiling when participants in [31] received the audio and visual signals (*mean* = 98.12% correct, *sd* = 2.63), and heard only the audio signal (*mean* = 96.88% correct; *sd* = 6.24), there was only a sufficient number of errors to examine in the visual only presentation condition (*mean* = 14.89% correct; *sd* = 17.71) of [31].

Several significant correlations provide additional justification for focusing on the errors from the visual-only presentation condition, the activation values from Simulation 3, and the 76 easy-hard CVC words selected above. First, the activation values of the easy-hard words obtained from the phonological nodes when only the viseme nodes were activated in the AV-net (Simulation 3) correlated positively with the accuracy rates of the human participants in [31] who viewed those same words (*r* = +.58, *p* < .001, *n* = 76), suggesting that the AV-net performs similarly to human participants. Second, the degree of the lexical equivalence class for the easy-hard words also correlated negatively with the accuracy rates of the human participants who viewed those same words (*r* = -.64, *p* < .001, *n* = 76). Related and third, the degree of the lexical equivalence class for the easy-hard words correlated negatively with the activation values of those words obtained from the AV-net (*r* = -.86, *p* < .001, *n* = 76). That is, words belonging to a lexical equivalence class that was confusable with many other lexical equivalence classes (via the substitution of a viseme, e.g., *beC* and *bes*) tended to be identified less accurately by human participants and in the AV-net.

The most frequently reported incorrect response for each of the 76 easy-hard CVC words in [31] was examined to determine if the error was in the same lexical equivalence class (LEC; i.e., it had the same visemes) as the easy-hard word that was visually presented, and if the error was a phonological neighbor (based on the 1 phoneme-metric) of the easy-hard word that was visually presented. If one considers it equally likely to make an error that results in a word that is in the same/different lexical equivalence class (LEC) as the word presented visually, then the (27 easy + 22 hard =) 49 errors that resulted in a word in a different LEC than the word that was presented is significantly more than the (11 easy + 16 hard =) 27 errors that resulted in a word in the same LEC as the word that was presented (χ^2^ = 6.37, *df* = 1, *p* = .01). Similarly, if one considers it equally likely to make an error that results in a word (not) being a phonological neighbor of the word presented visually, then the 53 errors that resulted in a word that was not a phonological neighbor of the word presented visually is significantly more than the 23 errors that resulted in a word that was a phonological neighbor of the word presented visually (χ^2^ = 11.84, *df* = 1, *p* < .001). These data are summarized in Table 3.

**Table 3:** Errors made in the visual only presentation condition in [31] for 76 easy-hard words.

**Easy Words**	**Different LEC**	27	**Phono neigh**	1
			**Not Phono neigh**	26
	**Same LEC**	11	**Phono neigh**	6
			**Not Phono neigh**	5
**Hard Words**	**Different LEC**	22	**Phono neigh**	6
			**Not Phono neigh**	16
	**Same LEC**	16	**Phono neigh**	10
			**Not Phono neigh**	6

Note. LEC = Lexical Equivalence Class. Phono neigh = phonological neighbor.

We further examined the 49 errors that resulted in a word in a different LEC than the word that was presented to determine if the addition, substitution, or deletion of a viseme was most common. We also examined the position in the word where the addition, substitution, or deletion tended to occur the most. Errors that involved a change of more than one viseme were classified as “Other.” These data are summarized Table 4.

**Table 4:** Type (and position) of 49 errors that resulted in a word in a different LEC made in the visual only presentation condition for the easy-hard words.

**Easy words**	**Addition**	2 (F)
	**Deletion**	1 (F)
	**Substitution**	6 (O)
		2 (V)
		7 (F)
	**Other**	9
**Hard words**	**Addition**	1 (F)
	**Deletion**	1 (F)
	**Substitution**	3 (O)
		2 (V)
		11 (F)
	**Other**	4

Note. Addition, Deletion, and Substitution = a change in a single viseme, O = onset position, V = medial vowel position, F = final position. Other = errors involving more than 1 viseme.

If one considers it equally likely for an Easy vs. Hard word to result in an error that is in a different lexical equivalence class (LEC), then the 27 errors that resulted in a word in a different LEC for the Easy words were comparable to the 22 errors that resulted in a word in a different LEC for the Hard words (χ^2^ = 0.51, *df* = 1, *p* = .48). Similarly, there was no difference in the number of errors that involved one viseme or more than one viseme for the Easy words, compared to the number of errors that involved one viseme or more than one viseme for the Hard words (χ^2^ = 1.43, *df* = 1, *p* = .23).

## Discussion

Network science has been used increasingly to understand how phonological, semantic, orthographic, or syntactic [44] relationships among words influence various language processes, including word-learning, speech perception, and speech production in healthy children and adults, as well as in individuals with developmental or acquired language disorders (for a review see [12]). Although much has been learned by using single-layer networks that represent only one type of relationship among words, the use of multilayer networks—where different types of information about words is represented in each layer—allows us to understand how different types of information interact during processing (e.g., [19]). In the present case, we created a multi-layer network to model the interaction of the phonological information that one hears with the visual information that one sees when a word is spoken [21–25].

We reported (in the S1 File) details about the structure of the AV-net, a multi-layer network linking phonological word forms to viseme representations of words. Measuring various aspects of the structure of a network is important because a central tenet of network science states that the structure of a network will influence how effectively and efficiently processes operate in the system [45, 46]. Although this multilayer network was constructed using the same similarity metric used in [7], namely the addition, deletion, or substitution of a single character (representing a phoneme or a viseme), the overall structure of this multilayer network differed in an important way from the single-layer network analyzed in [7]. Specifically, as shown in S1 Table of S1 File, the AV-net contained a single component, or group of nodes connected to each other in some way. In contrast, the network analyzed in [7] contained a giant component, several smaller components (containing nodes that were connected to each other, but not to other components or to the giant component), and many isolates (nodes not connected to anything).

The difference in the overall structure of the multi-layer network examined here compared to the single-layer network analyzed in [7]—despite using the same similarity metric in both cases—is important, because it weakens the claim that the structural characteristics observed in word-form networks “…arise primarily due to limitations in the kinds of networks generated by the one-step neighbor definition.” ([47] pg. 526; see also [48]; *cf*., [49]). This claim is further weakened by the emergence of similar structural characteristics in networks of phonological word-forms that do not use the one-step neighbor metric (e.g., [9]), and in networks that are technologic rather than linguistic in nature, and therefore do not represent phonological word-forms nor employ the one-step neighbor metric to connect related nodes [50]. Rather, the structural characteristics observed in various language networks (regardless of how they emerge) are not trivial and have important implications for language-related processes (for a review see [12]).

To examine how the structural characteristics of the AV-net might influence language-related processing, we performed several computer simulations using the diffusion of activation across the networks. We reiterate what has been stated elsewhere regarding the diffusion of activation across phonological networks, “…[it] should not be viewed as a proposal for a model of lexical processing. Rather, it is simply an exploration of how the structure of the phonological network might affect its function. . .” ([7] pg. 415). In the present case, our use of a multi-layer network also enabled us to explore how phonological information interacted with visual information (as one might obtain via lip-reading), making the network we constructed a useful tool to test training or other materials *in silico* before testing those materials further with human listeners/viewers.

Although we used only a subset of words (i.e., monosyllabic CVCs) to create the present network (see also [51]), the results of several analyses increase our confidence that the AV-net captures important characteristics about human performance under audio-only listening conditions, conditions where only visual information is available, and conditions in which audio and video information are present. Consider that native- and non-native listeners in psycholinguistic tasks identify more accurately auditorily presented lexically “easy” words than lexically “hard” words [52]. Pediatric users of cochlear implants also identify more accurately auditorily presented lexically “easy” words than lexically “hard” words [53]. Finally, [31] also observed that participants in all three conditions (audio-only, video-only, and AV) identified lexically “easy” words more accurately than lexically “hard” words.

To compare qualitatively the performance of the AV-net to the performance of human participants, we examined the activation levels of a selected set of 76 Easy-Hard words that were presented to the network. Higher activation levels after 5 time-steps in the network correspond to more accurate performance in human participants.

In Simulation 1, which simulated audio-video presentation in human participants by activating the viseme and phonological representations (but obtaining final activation values from only the phonological word nodes), we found that the network, like human participants, responded more accurately to the lexically “easy” words than to lexically “hard” words. In Simulation 2, which simulated audio-only presentation in human participants by activating only the phonological representations, we found that the network, like human participants, responded more accurately to the lexically “easy” words than to lexically “hard” words. An additional analysis comparing the final activation levels of the AV-net in Simulation 1 (where both viseme and phonological representations were activated) to the final activation levels of the AV-net in Simulation 2 (where only phonological representations were activated) demonstrated the often-reported boost in performance [22, 23] that the integration of visual and auditory information gives to word identification compared to relying on only auditory information. In Simulation 3, which simulated visual-only presentation in human participants by activating only the viseme representations, we found that the network, like human participants, responded more accurately to the lexically “easy” words than to lexically “hard” words.

Finally, in Simulation 4 we activated viseme nodes and obtained the final activation levels of viseme nodes in the "intact” AV-net and in the V-net (a single-layer network that had been “cut off” from phonological information). In the AV-net, which still had access to phonological information, we found that lexically “easy” words were responded to more accurately than lexically “hard” words. However, in the case of the V-net, which did not have access to phonological information, there was no difference in how accurately lexically “easy” and “hard” words were responded to in the network. The results of Simulation 4 suggest that the integration of auditory and visual information may not only support recognition of the auditory word form (as in [22, 23]), but may also support certain aspects of the visual recognition of spoken words (as in [24, 26]).

Our analyses of the errors made by human participants in the visual-only condition in [31] revealed several other novel, new, and unique discoveries, which may warrant future research. First, for the 76 easy-hard words we selected, human participants would sometimes get the lexical equivalence class correct, but then select the wrong phonological word-form in that LEC. However, human participants were more likely to get the LEC wrong, and then select from that incorrect pool of candidates the wrong phonological word-form. Second, when the wrong LEC was selected, the erroneous LEC tended to differ from the LEC of the word that was presented by the substitution of a single viseme. Additions and deletions of visemes occurred, as did errors that involved more than a single viseme, but these types of errors occurred less often. Together these error patterns suggest that future efforts devoted to the training of lip-reading may wish to focus on correctly identifying visemes in order to keep lip-readers in the correct LEC. Techniques commonly used in the training of novel phoneme categories for speakers of a second language [54] that rely on high-variability training with multiple speakers might be an approach worth considering in order to obtain experience with different faces, mouths, jaws, etc.

In addition, we made a novel observation: the degree of the lexical equivalence class (LEC) for the easy-hard words correlated negatively with the accuracy rates of the human participants who viewed those same words. The degree of the lexical equivalence class (LEC) for the easy-hard words also correlated negatively with the activation values of those words obtained from the AV-net. That is, a lexical equivalence class that was confusable with many other lexical equivalence classes (via the substitution of a viseme, e.g., *beC* and *bes*) tended to be identified less accurately by human participants and by the AV-net. Previous research found that the size of the LEC (i.e., the number of phonological word forms with the same visemes) influenced the accuracy of identifying spoken words from visual input alone [24, 25, 55]. The relationship we observed between the degree of the LEC and identification accuracy is a related, but novel discovery, which may warrant future research. The performance of the AV-net qualitatively resembling the performance of human participants in this novel finding increases our confidence that the AV-net is capturing something important about human behavior.

Finally, the incorrect phonological word-forms that were selected (whether the LEC was correct or not) tended to not be phonologically related to the word that was presented. This finding highlights that phonological and viseme information make unique contributions to perception and are not redundant pieces of information. This finding also points to future research that examines how other pieces of information might influence lip-reading. Perhaps future research could consider a multi-layer network that includes a layer with semantic information to examine how this additional source of “contextual” information might be used to help listeners resolve the competition among a set of phonological candidates (whether one is in the correct LEC or not).

In addition to the suggestions above for future research, researchers may also consider using feature-rich networks [56], which merges the structural characteristics of networks with vector-based information about individual words (e.g., word frequency, word length, part of speech, etc.). The structural information can be examined independent of the vector-based information, or the two types of information can be combined to reveal patterns that neither approach could reveal by themselves. A feature-rich multi-layer network also could be used to model the variation among speakers in their visual and auditory “intelligibility.”

Although the networks we examined in the present study have increased our understanding of visual speech perception (i.e., lip reading), and pointed to several directions for future research, there are a few limitations of the present work. First, we only examined English words. Much value will be gained by examining these phenomena in other languages [57]. Second, the words we used to construct the networks were restricted to monosyllabic CVCs. Including a wider range of words (i.e., different lengths, syllable structure, etc. [51]) will increase the utility of these networks, but we do not believe that expanding the networks will substantively change the results that we reported in the present study. Third, we only examined two layers in the AV-net. As described above, future research might consider networks with additional layers to better understand how various sources of information interact. Finally, although there is much evidence to suggest that phonological- and viseme-related information are integrated in the mental lexicon of humans [24, 25], other types of auditory information may also be involved in lip reading. For example, instead of phonological information, acoustic distance or acoustic absement might better discriminate among various lexical options [58].

Despite these limitations, we believe that the present study serves as a proof-of-concept for researchers interested in pushing research in visual speech perception in a new direction. Engineers may also benefit from the present work by incorporating visual information to improve automatic speech processing in face-to-face video (i.e., from videophone software). Designers of Augmentative and Alternative Communication (AAC) devices might also find value in the present work in developing devices that automatically read the lips of individuals with damaged vocal tracts. Finally, the present work could also find application in the field of biometric person identification to create methods to uniquely identify a specific person using face and voice input to grant them access to secure information or systems.

## Supporting information

S1 File (DOCX)

S1 Data (CSV)
