# Supplementary material for: Using network science to examine audio-visual speech perception with a multi-layer graph
Source: PLoS One. 2024 Mar 29;19(3):e0300926. doi: 10.1371/journal.pone.0300926 (PMC10980250; doi:10.1371/journal.pone.0300926)
Supplement: S1 File — (DOCX) [file pone.0300926.s001.docx]

**Supporting Information**

We used Gephi (0.9.2; [59]) and the *R* package qgraph [60] to measure various structural features of the AV-net. Table S1 summarizes the structural characteristics of the network.

Table S1. Summary data of the structural characteristics of the AV-net.

| **Network measure** | **AV-Net** |
| --- | --- |
| Nodes | 1,265 |
| Edges | 10,223 |
| Number of components | 1 |
| Average degree | 16.16 |
| Network Diameter | 6 |
| Average shortest path length | 3.3 |
| Average clustering coefficient | .33 |
| Number of communities (*Q*) | 10 (.56) |
| Small-worldness | 17.58 |

*Nodes* refers to the number of nodes in each network. *Edges* refers to the number of edges in each network.

A component in a network refers to a group of nodes that are all connected to each other in some way. *Number of components* refers to the number of such connected groups. In the present case, all of the nodes are somehow connected to each other, forming a single connected component.

Degree refers to the number of edges incident on a node. *Average degree* refers to the mean value of edges found for the nodes in a network. *Network Diameter* measures the shortest distance between the two most distant nodes in a network and is a rough estimate of how large a network is. In contrast, *Average shortest path length* is the mean value of the shortest path between all pairs of nodes in a network, and measures, on average, the number of edges that must be traversed to get from one node to another in the network.

The *clustering coefficient* measures the extent to which neighbors of a node are also neighbors of each other. More precisely, the clustering coefficient (*C*) is the ratio of the actual number of edges existing among neighbors of a given node to the number of all possible edges among neighbors if every neighbor was connected. *C* has a range from 0 to 1. When *C* = 0, none of the neighbors of a given node are neighbors of each other. When *C* = 1, the neighbors are fully interconnected, meaning every neighbor is also a neighbor of all the other neighbors of a given word. This variable (measured in a single-layer network of phonologically related words) has been shown to influence spoken word recognition [61], speech production [62], word-learning [63], long- and short-term memory [64], and perception of the speech to song illusion [65]. *Average clustering coefficient* is the mean clustering coefficient for the nodes in a network.

The average shortest path length and average clustering coefficient are used to determine if a network exhibits small-world characteristics. Typically, a small-world network has a short average path length and a high average clustering coefficient, which contribute to rapid navigation through the network compared to a network with the same number of nodes and edges that are connected in a different way. The current convention is to compute the small-worldness metric [66], with values greater than 1 (although a more conservative value is >=3) indicating that the network exhibits small-world characteristics.

Finally, we examined the community structure of the network using the Louvain community detection algorithm, a commonly used community detection algorithm [67]. *Communities* are smaller sub-groups of nodes that tend to be more connected to each other than to nodes found in another community (see [68]). Modularity, Q, is typically used to measure the extent to which clear, well-defined communities are found in a network [69]. Positive Q values close to the maximum of +1.0 indicate the presence of clear, well-defined communities in the network. The value of *Q* reported in Table 2 suggests that the network had relatively well-defined communities.

**References**

1. Bastian M, Heymann S, Jacomy M. Gephi: An open source software for exploring and manipulating networks. In Proceedings of the 3rd International AAAI Conference on Weblogs and Social Media; San Jose, CA, pp. 361–362. 2009.
2. Epskamp S, Cramer AOJ, Waldorp LJ, Schmittmann VD, Borsboom D. qgraph: Network Visualizations of Relationships in Psychometric Data. J Stat Softw. 2012; 48(4): 1–18. http://www.jstatsoft.org/v48/i04/.
3. Chan KY, Vitevitch MS. The Influence of the Phonological Neighborhood Clustering-Coefficient on Spoken Word Recognition. J Exp Psychol Human. 2009; 35: 1934-1949.
4. Chan KY, Vitevitch MS. Network structure influences speech production. Cognitive Sci. 2010; 34: 685–697.
5. Goldstein R, Vitevitch MS. The influence of clustering coefficient on word-learning: How groups of similar sounding words facilitate acquisition. Front Lang Sci. 2014; 5: 01307.
6. Vitevitch MS, Chan KY, Roodenrys S. Complex network structure influences processing in long-term and short-term memory. J Mem Lang. 2012; 67: 30-44.
7. Vitevitch MS, Ng JW, Hatley E, Castro N. Phonological but not semantic influences on the speech-to-song illusion. Q J Exp Psychol. 2021; 74: 585-597.
8. Humphries MD, Gurney K. Network "small-world-ness": a quantitative method for determining canonical network equivalence. PLOS ONE. 2008; 3(4): e0002051.
9. Girvan M, Newman MEJ. Community structure in social and biological networks. P Natl Acad Sci USA. 2002; 99: 7821–7826. doi: 10.1073/pnas.122653799
10. Siew CS. Community structure in the phonological network. Front Psychol. 2013; 4: 553.
11. Fortunato S. Community detection in graphs. Phys Rep. 2010; 486(3): 75–174.
